# Participation of pregnant women in a community-based nutrition program in Mumbai's informal settlements: Effect on exclusive breastfeeding practices

**DOI:** 10.1371/journal.pone.0195619

**Published:** 2018-04-05

**Authors:** Sheila Chanani, Anagha Waingankar, Neena Shah More, Shanti Pantvaidya, Armida Fernandez, Anuja Jayaraman

**Affiliations:** Society for Nutrition, Education and Health Action, Mumbai, India; Universidade de Sao Paulo, BRAZIL

## Abstract

**Background:**

In urban Maharashtra, India, approximately half of mothers exclusively breastfeed. For children residing in informal settlements of Mumbai, this study examines factors associated with exclusive breastfeeding, and whether exclusive breastfeeding, in a community-based nutrition program to prevent and treat wasting among children under age three, is associated with enrolment during the mother’s pregnancy.

**Methods:**

The nutrition program conducted a cross-sectional endline survey (October-December 2015) of caregivers in intervention areas. Factors associated with exclusive breastfeeding for infants under six months of age were explored using multi-level logistic regressions. Additionally, program surveillance data collected during home-based counselling visits documented breastfeeding practices for children under six months of age. Using the surveillance data (January 2014-March 2016), exclusive breastfeeding status was regressed adjusting for child, maternal and socioeconomic characteristics, and whether the child was enrolled in the program in utero or after birth.

**Results:**

The community-based endline survey included 888 mothers of infants. Mothers who received the nutrition program home visits or attended group counselling sessions were more likely to exclusively breastfeed (adjusted odds ratio 1.67, 95% CI 1.16, 2.41). Having a normal weight-for-height z-score (adjusted odds ratio 1.57, 95% CI 1.00, 2.45) was associated positively with exclusive breastfeeding. As expected, being an older infant aged three to five months (adjusted odds ratio 0.34, 95% CI 0.25, 0.48) and receiving a prelacteal feed after birth (adjusted odds ratio 0.57, 95% CI 0.41, 0.80) were associated with lower odds of exclusively breastfeeding. Surveillance data (N = 3420) indicate that infants enrolled in utero have significantly higher odds of being exclusively breastfed (adjusted odds ratio 1.55, 95% CI 1.30, 1.84) than infants enrolled after birth.

**Conclusions:**

Prenatal enrolment in community-based programs working on child nutrition in urban informal settlements of India can improve exclusive breastfeeding practices.

## Introduction

Globally, India has the largest number of malnourished children [1] and child deaths [2]. The 2011 census estimated that over 8 million children, aged zero to six years, are residing in informal settlements of India [3]. Children living in urban informal settlements experience low breastfeeding rates and high levels of malnutrition; they are vulnerable to gastrointestinal and other infections due to living in overcrowded spaces that lack adequate sanitation and waste management [4].

Benefits from exclusively breastfeeding infants under six months of age range from lower rates of infection and morbidity in infancy to longer term benefits in cognitive capacity [5]. Breastfeeding is a vital strategy in protecting young children from diarrhoea [6,7], a leading cause of poor nutritional status and death among infants and children [8]. In 2017, an estimated 99,499 child deaths due to diarrhoea and pneumonia in India could have been prevented through appropriate breastfeeding practices [9]. Thus, exclusive breastfeeding remains one of the most cost-effective methods for saving infants at large-scale [10–12].

Exclusive breastfeeding practices in urban areas are poorer than rural areas; in recent national surveys 52.1 percent of urban infants under six months of age were exclusively breastfed as compared with 56.0 percent of rural children [13]. In urban Maharashtra, exclusive breastfeeding rates are similarly low at 51.3 percent, as compared to 60.6 percent of rural children [14].

Most studies on exclusive breastfeeding in India use national-level data; there is little evidence focusing specifically on the needs of infants residing in urban informal settlements [15–19]. In these studies, factors associated with exclusive breastfeeding vary across regions and type of residency (urban versus rural). Associated factors for children include: age, gender, birth order, birthweight, current weight, antenatal care (ANC), breastfeeding promotion by a health worker, type and location of delivery, timing of first feed, use of prelacteal feeds, and immunization status. Associated maternal characteristics include: age, education, literacy, employment status, duration of pregnancy, birth intervals, and mother’s height. At the socioeconomic level, associated factors with exclusive breastfeeding include religion and economic status.

Community Management of Acute Malnutrition (CMAM) is a high-impact intervention that relies on community outreach to identify children with severe acute malnutrition (SAM), outpatient management of SAM cases without medical complications, and inpatient management of complicated cases [20]. Along with case-management of children screened as SAM or with moderate acute malnutrition (MAM), the nutrition program in this study expanded the scope of a typical CMAM approach to include a stronger prevention component by enrolling pregnant women and addressing feeding practices for all infants in the community under six months of age. This critical age group can be missed out in CMAM programs which focus on children aged six to 59 months of age [21]. The WHO recommends that child health interventions can optimally improve infant and child feeding practices through a 1000 day approach—targeting children from conception through the first two years of life [22]. The combined approach of prenatal and early-life intervention can better facilitate the promotion of appropriate infant feeding practices and prevent early abandonment of breastfeeding [23].

This study contributes to the limited evidence on delivery of breastfeeding and child nutrition interventions at scale [24–26] in India. Large-scale operational research on children from conception to two years of age is essential to achieve national child mortality targets [27]. The objectives of the study are: 1) to examine factors associated with exclusive breastfeeding practices among infants under six months of age residing in urban informal settlements of Mumbai, 2) to explore the effectiveness of enrolling pregnant women in a community-based child nutrition program on improving exclusive breastfeeding practices.

## Methods

### Study setting, program description and participants

Society for Nutrition, Education and Health Action (SNEHA), a Mumbai-based non-profit organization working in urban informal settlements, ran the adapted CMAM program from November 2011 through March 2016 in the urban informal settlements of Dharavi, Mumbai. Dharavi, one of the largest informal settlements in Asia, has an estimated population of 750,000 [28] to one million [29] residents. According to UN Habitat criteria, Dharavi is classified as a “slum” due to inadequate access to safe water and sanitation, poor structural quality of housing, and overcrowding and insecure residential status [29].

SNEHA implemented the nutrition program in partnership with Integrated Child Development Services (ICDS), the largest community-based government welfare program to monitor and support child growth in India. ICDS functions through a network of childcare centres called Anganwadi Centres—one centre per 1000 population provides non-formal preschool activities, supplementary nutrition, growth monitoring to track underweight children, health and nutrition education, and health referral services. The SNEHA nutrition program also collaborated with the Municipal Corporation of Greater Mumbai (MCGM), which has a network of local public health facilities that include tertiary hospitals, maternity hospitals, and community-level dispensaries and health posts.

The nutrition program covered 300 Anganwadi Centres, an estimated population of 300,000, which encompasses the entire ICDS administrative area of Dharavi. The program was delivered in phases—the first 150 centres served as pilot areas to test out varying iterations of the SNEHA model, and the last 150 centres functioned as the final model to be scaled and evaluated.

The primary goal of the program was a reduction in wasting prevalence among children under age three at the community-level. Prior to the baseline, SNEHA Community health workers (CHWs) worked in collaboration with ICDS to conduct a census house listing, identifying all pregnant women and children under age three in the intervention areas. SNEHA CHWs screened children for their wasting status and collected basic socioeconomic information. Throughout the intervention, SNEHA CHWs continuously identified new pregnancies, new migrant pregnant women, and missed pregnancies, as some women did not disclose their pregnancies until their second trimester. They also tracked newly married couples and couples with a single child who might be considering having a child.

**Fig 1:**
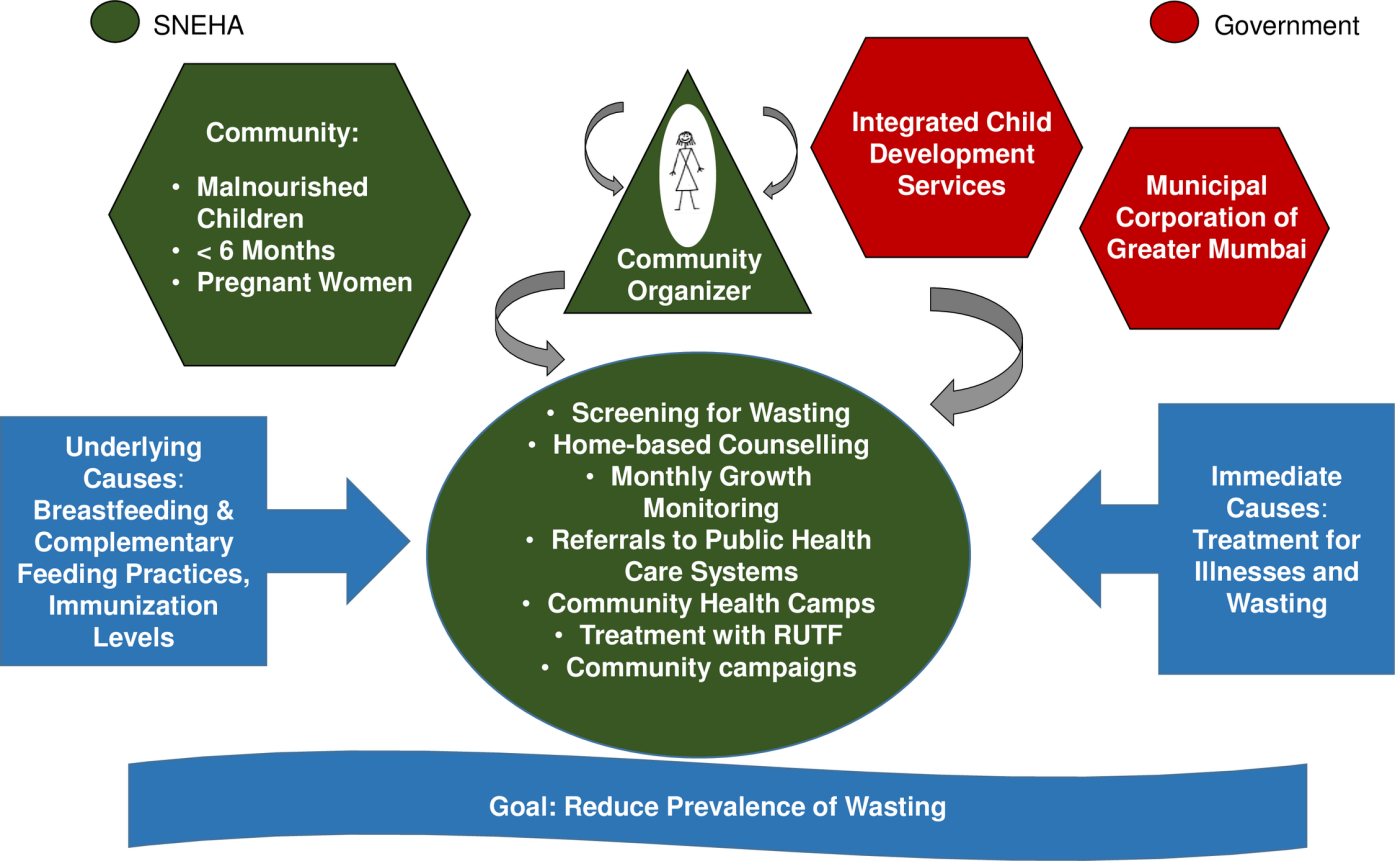
SNEHA program partnerships and key activities. Abbreviations: RUTF, Ready-to-use therapeutic food; SNEHA, Society for Nutrition, Education and Health Action.

Key activities of the nutrition program included: monthly growth monitoring conducted jointly with ICDS at the Anganwadi Centres; home-based counselling on feeding and care practices for priority groups (malnourished children, children under the age of six months, pregnant women); referrals to public health care facilities for treatment of illnesses and immunizations; access to medical assessments in community-based paediatric health camps; provision of antibiotics and locally produced ready-to-use food supplements; group activities for caregivers and community events for sensitization to child malnutrition; training of ICDS CHWs and supervisors to improve government service delivery; and convergence workshops and monthly meetings with ICDS and MCGM to improve coordination (Fig 1).

#### Program activities for promotion of exclusive breastfeeding

SNEHA CHWs provided home-based counselling visits to the pregnant women, with increasing frequency as the pregnancy advanced. During the home visits, SNEHA CHWs encouraged women to register for delivery and access early ANC. They provided information on the possible danger signs during pregnancy for which they should seek immediate medical care; these include leaking water, the baby not moving, continuous bleeding from vagina, severe abdominal pain prior to the eighth month, severe headaches and blurry vision, convulsions or loss of consciousness, fever, and oedema on their feet. SNEHA CHWs individually counselled women on preparation for institutional delivery, appropriate postnatal care, the importance of iron and folic acid for the baby’s growth, a nutritious diet, and rest.

A critical component of the counselling was to discuss the importance of breastfeeding practices such as initiation of breastfeeding within one hour of delivery, the benefits of colostrum, exclusive breastfeeding and avoidance of any prelacteal feeds. These monthly home-based counselling visits continued from pregnancy through the birth of the child until the child was six months old. Postpartum home visit frequency was higher for premature, low birthweight, and malnourished children. SNEHA CHWs counselled lactating mothers on breastfeeding, correct positioning, common problems and remedies, and the importance of exclusive breastfeeding. In addition to home visits, SNEHA CHWs also organized group meetings and baby shower events for pregnant women for peer sharing and learning.

### Data collection

To examine factors associated with exclusive breastfeeding practices this study uses the nutrition program endline evaluation data. SNEHA oversaw the implementation of a cross-sectional community-based survey across all 300 Anganwadi Centres from October through December 2015. Primary caregivers—typically mothers—of children under three were interviewed on: socioeconomic status (education, occupation, asset ownership, housing status); household and environmental sanitary conditions (water supply and treatment, toilet ownership); infant and young child feeding practices (IYCF); migration patterns; illness prevalence in children (diarrhoea and acute respiratory infections); child anthropometry (weight and height); and utilization of the SNEHA nutrition program and government services for maternal and child health (ICDS, MCGM). The 2012 Progress out of Poverty Index (PPI) was included to assess the likelihood that a household was living below specific poverty lines. The PPI incorporates household characteristics (number of children residing in household, type of fuel, father's education, and occupation) and asset ownership (10 small and large assets).

This study also examines active program surveillance data, collected by SNEHA CHWs, to assess the effectiveness of enrolling pregnant women in improving exclusive breastfeeding practices. At the time of enrolment of a pregnant woman, basic socioeconomic information were collected, along with her last date of menstruation and her access to health care services. Subsequently after the birth of the child, SNEHA CHWs made home-based counselling visits until the infant was six months of age and recorded information on the breastfeeding status of the child.

All data were collected in CommCare (Dimagi, USA), a mobile-based application designed for community health workers. The CommCare platform is open-source and enables data collection and storage on a web server using a standard mobile network. During the community-based endline survey, team supervisors reviewed all submissions before uploading to the server and conducted random crosschecks of information collected. For the surveillance data, SNEHA CHW supervisors crosschecked home visit submissions. Supervisors went to the homes of mothers to check on quality of counselling provided by the CHW and the accuracy of data submitted.

#### Sample size and sampling technique

Sampling for the community-based endline survey varied according to intervention phase. At the time of the evaluation, pilot intervention areas were receiving a limited intervention with reduced staff. These areas were staffed with approximately half the number of SNEHA CHWs to explore a transition of the nutrition program activities over to ICDS. Thus, those areas were sampled with the objective of getting prevalence estimates of wasting as an indicator of sustainability with the gradual handover to government partners.

The sample size for the scale-out intervention areas was set with the primary objective of measuring a 25 percent drop in the prevalence of wasting from baseline to endline. Assumptions for sample size calculations were 15 percent prevalence of wasting at baseline, intra-class correlation (ICC) (.04), alpha (.05) two-sided, and power (.80). For the scale-out intervention areas, each Anganwadi Centre had a target of 23 respondents for a total target sample of 3,450 respondents across 150 centres. In the pilot areas, each centre had a target of six or nine respondents, totalling a target sample of 1,040 respondents across 150 centres. Using the intervention house listing data, a household in each centre was randomly selected as the starting point to employ a modified systematic sampling approach. Investigators spun a pen to determine direction and continued to interview every third household within the boundaries of that centre service area until the target number of caregivers were identified and agreed to be interviewed [30]. If a caregiver had more than one child under three, the investigator collected information on the youngest child.

For the analysis of program surveillance data, home visit reports submitted by SNEHA CHWs, from January 2014 through March 2016, for infants under six months of age in the scale-out intervention areas are included. The reported breastfeeding practices from the most recent home visit given to each child was retained along with the total number of home visits received as of March 2016.

### Statistical analysis

Following a similar analytical strategy as employed by Setegn et al. [31] and Hunegnaw et al. [32], logistic regression models were estimated to examine associations of the dependent variable, exclusive breastfeeding, with child, mother and socioeconomic characteristics. Multi-level random intercept logistic regressions were estimated controlling for correlation within the Anganwadi Centre clusters. For each characteristic included in the multivariable models the crude odds ratio are presented along with the adjusted odds ratio (AOR) model and 95% confidence intervals (CI). All analyses were conducted in STATA 15.0 (StataCorp, College Station, TX).

#### Dependent variable

In the community-based endline survey, the outcome of exclusive breastfeeding was measured through a series of questions asked to the mother on what the infant had consumed in the previous 24 hours. Children were coded as exclusively breastfeeding if they reported currently (based on the 24-hour recall period) drinking breastmilk, no plain water, no infant formula, no other form of milk, no yoghurt-based drinks (eg *lassi* or *chaas*), no fruit juice, no clear broth, no tea or coffee, no soda, and no other liquids, solids or semi-solids. Any child that reported zero night and day feeds of breastmilk was coded as not exclusively breastfed.

In the surveillance data children were coded as exclusively breastfeeding if they reported currently (based on the 24-hour recall period) drinking breastmilk, no other milk or milk-based products, no other liquids, and no other solids or semi-solids.

#### Independent variables

For the regression models using the community-based endline survey, bivariate associations of exclusive breastfeeding with independent variables were explored using Pearson chi-square for categorical variables. Comparison tests were adjusted for the cluster-survey design using the STATA “svy” commands. ICC coefficients were calculated using STATA post-estimation commands.

Independent variables included in the final regression model using the community-based survey data had a p-value less than 0.20 in the bivariate analysis. These include: child age; gender; birth order; weight-for-height (wasting) nutritional status; whether the infant was delivered in Mumbai or outside the city; whether the infant was given a prelacteal feed; whether the mother received a SNEHA home visit or attended a SNEHA group session; location of antenatal care (ANC) (No ANC, Private Facility, Public Facility); type of birth facility (Home, Public Facility, Private Facility); mother’s employment status; and household PPI (poverty index) likelihood score. Additional explanatory variables from the literature on determinants of exclusive breastfeeding in India (advice from a community health worker, maternal education, maternal body mass index, home ownership, and religion) were also included in the final model.

For the surveillance data regression model, independent variables included basic socioeconomic variables collected for each pregnant woman or child at the time of screening, along with a dummy variable for children whose mothers enrolled into the program while the child was in utero.

A child is wasted if her weight-for-height Z-score (WHZ) is less than two standard deviations (SD) below the median of the WHO 2006 growth standards reference population and overweight if her WHZ is greater than two SD above the median. Normal WHZ status is less than or equal to 2 SD above the median and greater than or equal to -2 SD above the median. WHZ was calculated using Emergency Nutrition Assessment (ENA) software. WHZ for children with outlier values (> 5 SD or < −5 SD) or significant discrepancies in their two length or weight measurements were set to missing. Public health facilities include municipal maternity hospitals, tertiary hospitals, and health posts. Private facilities include private hospitals, private medical doctors, and any other non-government individual medical practitioner (licensed and unlicensed).

### Ethical statement

The study received ethical approval from the Bandra Holy Family Medical Research Society, Mumbai. Respondents in the community-based endline survey gave their written informed consent prior to participation. Surveillance data presented are part of implementation data and the ethics committee approved use of this data to study program impact and effectiveness.

## Results

Across the 300 Anganwadi Centres, 4,527 caregivers with children under age three were sampled during the community-based survey conducted between October and December 2015. Of these 888 (20 percent) were mothers with a child under the age of six months. In the sample of children under six months of age, 64.4 (95% CI 61.0, 67.8) percent were exclusively breastfeeding.

Table 1 presents descriptive frequencies and proportions for characteristics of the respondents and exclusive breastfeeding rates for those characteristics. Approximately 96 percent of mothers reported that they were not working for income and 14.5 percent were underweight with a body mass index (BMI) lower than 18.5. Over 18 percent of the mothers reported they were illiterate or had not received any education beyond the 5^th^ grade. Nearly 13 percent of the infants were wasted Table 1.

**Table 1:** Child, maternal, and socioeconomic characteristics and rates of exclusive breastfeeding by characteristic of infants under six months of age in informal settlements of Mumbai, India.

	All children less than 6 months of age		Exclusive Breastfeeding—Yes	
	N = 888	%	N = 572	%
***Child Characteristics***				
**Exclusive Breastfeeding**				
** Yes**	572	64.4		
** No**	316	35.6		
**Age**				
** Less than 3 months**	461	51.9	347	75.3***
** 3–5 months**	427	48.1	225	52.7
**Gender**				
** Female**	424	47.8	290	68.4*
** Male**	464	52.3	282	60.8
**Birth Order**				
** First**	326	36.7	198	60.7
** Second**	322	36.3	211	65.5
** Third**	151	17.0	104	68.9
** Fourth and above**	89	10.0	59	66.3
**Weight for Height**				
** Wasting (< -2SD)**	112	12.7	64	57.1
** Normal (-2SD to 2SD)**	754	85.6	494	65.5
** Overweight (> 2SD)**	15	1.7	10	66.7
**Child Born in Mumbai**				
** Yes**	764	86.6	506	66.2*
** No**	118	13.4	66	55.9
**Prelacteal Feed Given**				
** Yes**	298	33.8	163	54.7***
** No**	584	66.2	409	70.0
***Maternal Characteristics***				
**Breastfeeding Advice from ICDS CHW in Past Month**				
** Yes**	179	20.2	121	67.6
** No**	709	79.8	451	63.6
**Received SNEHA Home Visit or Attended SNEHA Group Session**				
** Yes**	659	74.2	440	66.8*
** No**	229	25.8	132	57.6
**Location of ANC**				
** No ANC**	19	2.2	12	63.2***
** Public Facility**	475	53.9	335	70.5
** Private Facility**	388	44.0	225	58.0
**Type of Birth Facility**				
** Home**	26	3.0	15	57.7***
** Public Facility**	522	59.3	374	71.7
** Private Facility**	333	37.8	183	55.0
**Maternal Education**				
** Illiterate, Primary, Informal**	167	18.8	110	65.9
** Secondary (5–10 grade)**	493	55.5	323	65.5
** Higher secondary (11 grade & higher)**	228	25.7	139	61.0
**Maternal Working Status**				
** Not working**	854	96.2	554	64.9
** Working**	34	3.8	18	52.9
**Maternal BMI**				
** Underweight (<18.5)**	127	14.5	85	66.9
** Healthy (18.5 to 25)**	489	55.9	325	66.5
** Overweight (>25)**	259	29.6	157	60.6
***Socioeconomic Characteristics***				
**Home Ownership**				
** Yes**	520	58.6	335	64.4
** No**	368	41.4	237	64.4
**PPI: Likelihood Below the $2.16/day/PPP Line**				
** Less than 65%**	237	26.8	143	60.3
** 65 up to 75%**	161	18.2	98	60.9
** 75 up to 85%**	326	36.8	216	66.3
** 85 up to 99%**	161	18.2	115	71.4
**Religion**				
** Muslim**	404	45.5	259	64.1
** Hindu**	433	48.8	283	65.4
** Other**	51	5.7	30	58.8

ANC, Antenatal Care; BMI, Body Mass Index; CHW, Community Health Worker; ICDS, Integrated Child Development Services; PPI, Progress out of Poverty Index; PPP, Purchasing Power Parity; SD, Standard Deviation; SNEHA, Society for Nutrition, Education and Health Action. Pearson chi-square comparing exclusive breastfeeding status by characteristic

*P-value: ≤0.05

*** P-value: ≤0.001.

Younger infants less than three months of age had higher levels of exclusive breastfeeding (75.3 percent) as compared with the older infants aged three to five months (52.7 percent). Female infants also had higher levels of exclusive breastfeeding (68.4 percent) as compared with male infants (60.8 percent). Approximately 87 percent of the infants were born in Mumbai, and they reported higher rates of exclusive breastfeeding (66.2 percent) as compared to those born outside the city (55.9 percent). Nearly thirty-four percent of all mothers reported giving some form of prelacteal liquid in the first days after birth that was not breastmilk—those who gave prelacteals had lower levels of exclusive breastfeeding (54.7 percent) as compared to those who did not give any (70 percent). Approximately 20 percent of mothers received breastfeeding advice from a government health worker, and 74.2 percent of sampled mothers received home visits by a SNEHA CHW or attended a SNEHA group session. Mothers who reported getting these services from SNEHA had higher exclusive breastfeeding rates as compared with those who did not receive them (66.8 percent versus 57.6 percent).

While the private sector plays an important role in urban informal settlements, women still sought ANC (53.9 percent) and delivered their babies (59.3 percent) in public health facilities. Receiving ANC in a public facility is associated with higher levels of exclusive breastfeeding (70.5 percent) as compared with private sector facilities (58.0 percent). Similarly, giving birth in a public facility is associated with higher rates of exclusive breastfeeding (71.7 percent) as compared to mothers who gave birth in private facilities (55.0 percent).

Multi-level logistic regressions based on endline data indicate that having a normal weight-for-height status (AOR 1.57, 95% CI 1.00, 2.45), as compared with being wasted, and receiving a home visit from a SNEHA CHW or attending a SNEHA group session (AOR 1.67, 95% CI 1.16, 2.41) were associated with significantly higher odds of exclusive breastfeeding status. Being an older infant aged three to five months (AOR 0.34, 95% CI 0.25, 0.48), as compared to less than three months old, and receiving a prelacteal feed (AOR 0.57, 95% CI 0.41, 0.80) were significantly associated with lower odds of exclusively breastfeeding Table 2. The ICC for the AOR model is 0.04, indicating a small clustering effect at the Anganwadi Centre level; this falls within the range observed in a community-based trial to improve maternal and child health in other informal settlements of Mumbai [33].

**Table 2:** Factors associated with exclusive breastfeeding for children under six months of age residing in informal settlements of Mumbai, India.

(N = 888)	Crude Odds Ratio (95% CI)	Adjusted Odds Ratio^a^ (95% CI)
***Child Characteristics***		
**Child Age (3 to 5 months)**	0.35 (0.26–0.47)	0.34 (0.25–0.48)***
**Child Gender (Female)**	1.44 (1.07–1.92)	1.33 (0.97–1.82)
**Birth Order**		
** First**	1.00	1.00
** Second**	1.23 (0.88–1.71)	1.17 (0.81–1.70)
** Third**	1.41 (0.92–2.17)	1.21 (0.72–2.01)
** Fourth and Above**	1.28 (0.77–2.13)	1.23 (0.64–2.35)
**Weight for Height**		
** Wasting (< -2SD)**	1.00	1.00
** Normal (-2SD to 2SD)**	1.45 (0.95–2.21)	1.57 (1.00–2.45)*
** Overweight (> 2SD)**	1.48 (0.45–4.81)	2.28 (0.60–8.66)
**Child Born in Mumbai**	1.63 (1.07–2.47)	1.12 (0.69–1.82)
**Prelacteal Feed Given**	0.52 (0.38–0.70)	0.57 (0.41–0.80)***
***Maternal Characteristics***		
**Breastfeeding Advice from ICDS CHW in Past Month**	1.17 (0.81–1.68)	1.04 (0.69–1.56)
**Received SNEHA Home Visit or Attended SNEHA Group Session**	1.50 (1.08–2.07)	1.67 (1.16–2.41)**
**Location of ANC**		
** No ANC**	1.00	1.00
** Public**	1.33 (0.49–3.60)	0.57 (0.18–1.75)
** Private**	0.75 (0.27–2.03)	0.64 (0.21–1.98)
**Location of Birth**		
** Home**	1.00	1.00
** Public**	1.85 (0.81–4.25)	1.42 (0.53–3.80)
** Private**	0.88 (0.38–2.05)	0.67 (0.24–1.86)
**Maternal Education**		
** Illiterate, Primary, Informal**	1.00	1.00
** Secondary (5–10 grade)**	0.97 (0.66–1.43)	0.98 (0.64–1.50)
** Higher secondary (11 grade & higher)**	0.80 (0.52–1.24)	1.15 (0.68–1.96)
**Maternal Working Status—Not Working**	1.66 (0.81–3.42)	1.11 (0.46–2.64)
**Maternal BMI**		
** Underweight (<18.5)**	1.00	1.00
** Healthy (18.5 to 25)**	0.99 (0.64–1.53)	0.87 (0.55–1.40)
** Overweight (>25)**	0.77 (0.48–1.22)	0.68 (0.41–1.15)
***Socioeconomic Characteristics***		
**Home Ownership**	1.00 (0.75–1.34)	1.05 (0.75–1.47)
**PPI: Likelihood Below the $2.16/day/PPP Line**	2.65 (1.17–6.03)	1.17 (0.38–3.62)
**Religion**		
** Muslim**	1.00	1.00
** Hindu**	1.05 (0.77–1.42)	1.04 (0.74–1.47)
** Other**	0.81 (0.43–1.52)	0.96 (0.48–1.94)
**Intra-Class Correlation**	Null (.06)	0.04

ANC, Antenatal Care; BMI, Body Mass Index; CHW, Community Health Worker; ICDS, Integrated Child Development Services; PPI, Progress out of Poverty Index; PPP, Purchasing Power Parity; SD, Standard Deviation; SNEHA, Society for Nutrition, Education and Health Action. Level of significance for adjusted model

*P-value: ≤0.05

**P-value: ≤0.01

*** P-value: ≤0.001.

^a^Adjusted model contains all variables in table (N = 869). Total sample size falls due to missing values generated primarily in removing WHZ outliers.

Program participants' characteristics from the program surveillance data are compared for children under six months of age monitored in utero to those enrolled into the program after birth. For all 3420 infants monitored in the program during the period of study, their last recorded breastfeeding status indicated that 70.3 percent (95% CI 67.2, 73.4) were exclusively breastfeeding. For infants enrolled in utero 76.0 percent (95% CI 72.5, 79.4) were exclusively breastfeeding as compared with 66.3 percent (95% CI 62.8, 70.0) for infants enrolled after birth. The children enrolled after birth are older, with approximately 95 percent in the three to five month age group, than children enrolled in utero, with 85 percent in the three to five month age group. Infants enrolled in utero also come from households with higher levels of home ownership (58.1 percent as compared with 53.8 percent) and lower likelihoods of poverty; 17.5 percent are in the highest likelihood range of poverty as compared with 29.4 percent for those enrolled after birth Table 3.

**Table 3:** Child, maternal, and socioeconomic characteristics of infants by enrolment status in a community-based child nutrition program in informal settlements of Mumbai, India.

Characteristics	All children less than 6 months of age (N = 3420)		Enrolled after Birth (N = 2006)		Enrolled in Utero (N = 1414)	
	N	%	N	%	N	%
**Exclusive Breastfeeding**						
** Yes**	2,404	70.3	1,330	66.3	1,074	76.0***
** No**	1,016	29.7	676	33.7	340	24.0
**Child Age**						
** Less than 3 months**	314	9.2	102	5.1	212	15.0***
** 3 to 5 months**	3,106	90.8	1,904	94.9	1,202	85.0
**Child Gender**						
** Female**	1,713	50.1	1,017	50.7	696	49.2
** Male**	1,707	49.9	989	49.3	718	50.8
**Maternal Working Status**						
** Not Working**	3,290	96.3	1,937	96.7	1,353	95.7
** Working**	128	3.7	67	3.3	61	4.3
**Home Ownership**						
** Yes**	1,901	55.6	1,079	53.8	822	58.1*
** No**	1,517	44.4	925	46.2	592	41.9
**PPI: Likelihood Below the $2.16/day/PPP line**						
** Less than 65%**	693	20.4	334	16.8	359	25.5***
** 65 up to 75%**	703	20.7	351	17.6	352	25.0
** 75 up to 85%**	1,174	34.5	722	36.2	452	32.1
** 85 up to 99%**	834	24.5	587	29.4	247	17.5

PPI, Progress out of Poverty Index; PPP, Purchasing Power Parity. Pearson chi-square for categorical variables comparing children screened in utero to children screened after birth

*P-value: ≤0.05

*** P-value: ≤0.001.

The logistic regression analyses from the surveillance data indicates that after adjusting for differences between the two groups, infants enrolled in utero have a significantly higher odds of being exclusively breastfed (AOR 1.55 95% CI 1.30, 1.84). Older infants three to five months of age were associated with lower odds of exclusive breastfeeding (AOR 0.17 95% CI 0.11, 0.27) as compared with infants less than three months of age Table 4. The ICC for the AOR model was 0.18, indicating that there was a stronger clustering effect at the Anganwadi level among the monitored participants data as compared with the community-based sample.

**Table 4:** Association between infant enrolled during pregnancy and exclusive breastfeeding for participants in a community-based child nutrition program in informal settlements of Mumbai, India.

Characteristics (N = 3420)	Crude Odds Ratio (95% CI)	Adjusted Odds Ratio^a^ (95% CI)
**Enrolled During Pregnancy**	1.69 (1.43–2.00)	1.55 (1.30–1.84)***
**Child Age (3 to 5 months)**	0.15 (0.10–0.23)	0.17 (0.11–0.27)***
**Child Gender–Female**	0.87 (0.75–1.03)	0.89 (0.75–1.05)
**Maternal Working Status—Not Working**	1.08 (0.71–1.64)	1.10 (0.72–1.70)
**Home Ownership**	0.86 (0.73–1.01)	0.89 (0.75–1.06)
**PPI: Likelihood Below the $2.16/day/PPP Line**	1.4 (0.85–2.31)	1.57 (0.92–2.68)
**Intra-Class Correlation**	Null (0.17)	0.18

PPI, Progress out of Poverty Index; PPP, Purchasing Power Parity. Level of significance for adjusted model

***P-value: ≤0.001.

^a^Adjusted Model contains all variables in the table. Sample size falls from 3420 to 3404 due to missing values.

## Discussion

This large-scale child nutrition program was associated with improved wasting levels in urban informal settlements of Mumbai, and among secondary indicators, higher levels of exclusive breastfeeding [34]. This study aimed to explore activities that could have contributed to the improvement in exclusive breastfeeding practices.

In our sample, a normal weight-for-height status (as compared with wasting) and exclusive breastfeeding were positively associated. A systematic review of 35 studies found that the relationship between breastfeeding promotion and growth was not conclusive [35]. In a similar context, Dhaka, Bangladesh, exclusive breastfeeding on low birthweight babies led to improved weight and length gains [36]. In this study, approximately 23 percent of the infants in the community-based endline sample were born low birthweight and 7 percent had experienced diarrhoea in the previous two weeks (not shown in tables)—exclusive breastfeeding is an available strategy to achieve normal growth in spite of these disadvantages.

The results of the community-based model identified an inverse association between prelacteal supplementation with exclusive breastfeeding. Evidence gathered by the WHO indicates that use of prelacteal supplements without any medical indication is associated with early termination of breastfeeding [37]. Among Hindu and Muslim families, provision of prelacteal feeds can be a traditional ceremonial practice that includes a range of items such as honey and clarified butter [38]. The community-based endline data indicated that among the mothers who reported giving prelacteal feeds, 63 percent gave milk, 35 percent gave formula, and 27 percent gave honey (not shown in tables). Thus, counselling mothers on infant feeding practices after those first few critical days after birth have already passed is a missed opportunity for child health programs. Identifying women during pregnancy can support mothers and family members in having appropriate information on the risks of providing prelacteal supplementation.

The community-based model indicates a positive association for the SNEHA nutrition program in improving exclusive breastfeeding practices. Mothers were significantly more likely to report exclusively breastfeeding their infants if they had receiving counselling services from SNEHA or attended a SNEHA group session. Children enrolled in utero in the SNEHA program benefited from receiving counselling services earlier and more frequently. The program surveillance data indicated that infants enrolled in utero received an average of nine (median = eight) home visits from a SNEHA CHW and children enrolled after birth received an average of five (median = four) home visits. The average and median age that a child entered the program if the mother was enrolled post-pregnancy was 2.8 months of age.

A qualitative assessment of the SNEHA nutrition program found that the key contribution to the overall success of the program was the continuous presence of trusted and informed CHWs [34]. During the home visits, SNEHA CHWs individually responded to mother’s breastfeeding concerns in a timely manner. Approximately 11 percent of mothers reported a problem with breastfeeding at the time of the home visit, with the majority reporting an issue of “low milk supply”. The SNEHA CHW was equipped with specific strategies for dealing with this issue, and trained to provide the reassurance and confidence that the mother could continue to breastfeed exclusively.

Other studies and systematic reviews also report the importance of breastfeeding counselling in the third trimester to provide education, support intentions for breastfeeding, and build mothers’ confidence to breastfeed [39]—exclusive breastfeeding is affected by both prenatal and postpartum management [40,41]. Similar community-based interventions with vulnerable populations in Dhaka, the United States, Mexico, Burkina Faso, and Uganda found that prenatal and postnatal counselling at home improved exclusive breastfeeding practices [42–45]. Similar to the findings of the SNEHA program, these interventions support the benefits of early and repeated contact from a trusted counsellor. Additionally, the multi-pronged nature of the SNEHA nutrition program, by working with available government infrastructure, organizing community-based group meetings, and providing counselling at home, likely also contributed to the improvement in breastfeeding practices [46,47].

### Limitations

One of the key limitations is the limited number of variables available in the surveillance data to adjust for confounding factors in the multivariable regression analysis. Another limitation is the migratory movement common to urban informal settlements—approximately 13 percent of infants were not born in Mumbai. There may be other differences among the infants under six months of age who migrated to Mumbai during infancy for which the surveillance data analysis has not adjusted due to lack of data.

The use of a 24 hour/current status recall methodology is known to overestimate exclusive breastfeeding practices [48]. Self-reporting of breastfeeding practices during the home visits may be positively biased; however, this bias would be similar across all mothers in the surveillance data. Finally, given that the study uses cross-sectional data, the models merely indicate association and not causal link between exclusive breastfeeding status and independent variables.

## Conclusion

Factors associated with exclusive breastfeeding in urban informal settlements of Mumbai indicate a positive relationship with normal weight-for-height anthropometric status, counselling from a community health worker, and a negative association with giving prelacteal feeds. The program surveillance data provides clear evidence of a positive association with enrolment of children while in utero and exclusive breastfeeding. This study provides more evidence in support of prenatal intervention in child nutrition programs—to the best of our knowledge, this is the only study in the current literature examining how child nutrition programs in urban informal settlements of India can integrate pregnant women to improve exclusive breastfeeding outcomes.

## Supporting information

S1 Dataset Community-based endline dataset. (DTA)

S2 Dataset Nutrition program surveillance data for home visits of infants under six months of age. (DTA)

S3 Dataset 2012 Progress out of Poverty scores and likelihoods. (DTA)

S1 File Community-based endline survey questions. (XLSX)

S2 File Nutrition program surveillance questions for home visits of infants under six months of age. (XLSX)
